# Multimechanistic Single-Entity Combinations for Chronic Pain Control: A Narrative Review

**DOI:** 10.7759/cureus.26000

**Published:** 2022-06-16

**Authors:** Joseph Pergolizzi, Peter Magnusson, Flaminia Coluzzi, Frank Breve, Jo Ann K LeQuang, Giustino Varrassi

**Affiliations:** 1 Cardiology, Native Cardio Inc., Naples, USA; 2 Cardiology, Center of Research and Development Region Gävleborg/Uppsala University, Gävle, SWE; 3 Medicine, Cardiology Research Unit, Karolinska Institutet, Stockholm, SWE; 4 Medical and Surgical Sciences, Sapienza University of Rome, Rome, ITA; 5 Department of Pharmacy, Temple University, Philadelphia, USA; 6 Scientific Communication, NEMA Research, Inc., Naples, USA; 7 Research, Paolo Procacci Foundation, Rome, ITA

**Keywords:** pharmacology, chronic pain, pain control, analgesic, opioids

## Abstract

Atypical opioids such as tramadol, tapentadol, and cebranopadol combine two complementary mechanisms of action into a single molecule, creating novel analgesic agents. These are synthetic small molecules: cebranopadol is not yet market released; tramadol and tapentadol are commercially available and have immediate-release (IR) and extended-release (ER) formulations. Tramadol has been widely used in the United States in recent years and works as a prodrug in that its metabolites are active in inhibiting serotonin and norepinephrine reuptake. Tapentadol is a direct-acting agent with a faster onset of action and is a mu-opioid-receptor agonist and also inhibits noradrenaline reuptake. Cebranopadol is the newest of these drugs, a first-in-class atypical analgesic that combines mu-opioid receptor (MOR) agonism with activity at the nociception/orphanin (NOP) FQ petide receptors. Cebranopadol may be considered a partial kappa-opioid receptor agonist as well. The pharmacology of these unique single-entity agents allows them to offer analgesic benefit with fewer side effects and risks. Clinical studies have demonstrated the safety and efficacy of tramadol and tapentadol, and promising but limited studies for cebranopadol show good analgesic effect and safety. Serotonin toxicity or ‘serotonin syndrome’ may occur with accumulation of serotonin with tramadol. While the misuse of these agents is limited in the United States, tramadol misuse is prevalent in Iran and parts of Africa. Patients have been successfully rotated from one of these agents to another. All three agents show promise in the treatment of cancer and non-cancer pain and their unique formulation in a single molecule reduces the pill burden.

## Introduction and background

The tandem public health crises of opioid overdose and the chronic pain have led to the current “re-imagining” of long-term analgesic therapy. Despite the fact that pain is one of the most ancient complaints in medicine, the perfect analgesic agent has yet to be developed. Opioids are effective analgesics that act as agonists of the opioid receptors, but by activating these pathways, they can cause various potentially treatment-limiting side effects, such as respiratory depression, somnolence, dizziness, opioid-induced constipation, nausea, vomiting, pruritus, opioid dependence, and opioid use disorder (OUD). The increasing prevalence of OUD has heightened scrutiny on opioid prescribing overall, leaving drug developers and healthcare professionals to search for safer analgesic therapy without sacrificing effectiveness.

The recent development of atypical opioids is a partial response to that challenge [1]. Borrowing from the principals of multimechanistic analgesia where two or more agents with complementary and synergistic mechanisms of action are utilized, a new category of analgesic agents offers dual mechanisms of action in a single molecule. There are a number of atypical opioids, perhaps the best known of which is buprenorphine, which is active at multiple opioid receptors. This review will discuss tramadol, tapentadol, and cebranopadol, which may be viewed as a progression of atypical single-entity opioids with dual mechanisms of action. However, these three agents have some important distinctions.

The aim of this narrative review is to evaluate recent studies of these agents and whether these agents may be considered safe, effective pain relievers in the population of chronic cancer and non-cancer pain patients, for whom there are often no ideal analgesic agents. The objective is to determine if they present specific advantages or disadvantages to these patients with their unique and atypical mechanisms of action.

## Review

The agents

These three atypical opioids are synthetic small molecules offering mu-opioid receptor (MOR) agonism in combination with additional synergistic effects. All are available as oral analgesics and the two commercially available products, tramadol and tapentadol, are offered in immediate-release (IR) and extended-release (ER) formulations. Cebranopadol has not yet been commercially released in the market.

Tramadol, the oldest of these three agents, has become widely used in the United States for pain control, with about 36.5 million prescriptions dispensed in the United States in 2018 [2]. The analgesic effect of tramadol resides in its active metabolites, making it a prodrug [3]. The two pharmacologically active enantiomers of tramadol each have active metabolites of their own: (+)-O-desmethyl-tramadol, (+)- tramadol, and (-)- tramadol. The first two of these metabolites are MOR agonists and (+)-tramadol also inhibits serotonin reuptake, while (-)- tramadol inhibits norepinephrine uptake [4]. The Drug Enforcement Administration (DEA) lists tramadol as a Schedule IV controlled substance.

Developed in the late 1980s, tapentadol is sometimes unfairly portrayed as a newer version of tramadol [5]. Unlike tramadol, tapentadol has one enantiomer and is a direct-acting agent rather than a prodrug. Unlike tramadol, tapentadol is not metabolized by the cytochrome-P (CYP) 450 enzymes [6]. Chemically, tapentadol differs from tramadol in that tapentadol has opened the cyclohexane ring and replaced the tertiary hydroxyl group. It has a more rapid onset of action than tramadol and offers greater nociceptive pain control [6]. Like tramadol, it combines dual mechanisms of action in a single molecule: agonistic activity at the MORs and inhibition of noradrenaline reuptake [7]. Tapentadol selectively binds to the MORs but with an affinity about 44 times less than that of morphine, yet offering analgesia about two to three times less than that of morphine because of its noradrenergic activities [8]. According to the DEA, tapentadol is a Schedule II controlled substance.

The dual mechanisms of action of cebranopadol, a first-in-class atypical analgesic, combines MOR agonism along with agnostic activity at the nociception/orphanin (NOP) FQ peptide receptors [9,10]. The NOP/FQ receptors are the most recently discovered opioid receptors, representing the fourth such family along with the mu, kappa, and delta receptors [11]. Cebranopadol may also be considered a partial agonist at the kappa-opioid receptors [12]. At a molecular level, NOP is a 17-amino-acid polypeptide that is similar to typical opioids [9]. The pharmacological activity of cebranopadol differs from those of typical opioids, and, for that reason, may be more tolerable and confers less risk of respiratory depression [9]. Once-daily administration of cebranopadol is possible in a small tablet, reducing the pill burden [13]. Since cebranopadol is not yet approved for market release, it has not yet been scheduled by the DEA as a controlled substance,

Efficacy for chronic pain

Tramadol

Tramadol may be described as a centrally acting analgesic and a weak opioid, and there is evidence in the literature that it may confers effective pain relief for some patients [14]. Tramadol may be considered a first-line treatment for musculoskeletal pain syndromes [15]. An ER formulation was found to be significantly more effective than placebo at reducing the moderate to severe chronic pain associated with osteoarthritis [16]. However, many studies of tramadol are small. In a systematic review and meta-analysis of six trials using tramadol for the treatment of chronic neuropathic pain, the number needed to treat (NNT) in order to achieve ≥50% pain control was 4.4 (95% CI, 2.9 to 8.8) but there is only modest evidence that tramadol confers an analgesic benefit in chronic neuropathic pain syndromes [17]. In fact, systematic reviews and meta-analyses have found little high-quality evidence to support the role of tramadol monotherapy or tramadol in combination with acetaminophen for osteoarthritis [18] or chronic low back pain (cLBP) [19], although tramadol was rated better than placebo for pain control in cLBP. In a systematic review and meta-analysis (two studies, n=672) it was found that weak opioids, including but not limited to tramadol, may be effective analgesics for some rheumatoid arthritis patients, but these agents may have potentially treatment-limiting side effects [20].

There is only limited and low-quality evidence for the use of tramadol in adult cancer pain patients [21]. In fact, the role of tramadol as a weak opioid in oncologic pain control is unclear [22], and tramadol, like other opioids, may have undesirable effects on immune cells [23]. Further research is needed, as these effects appear to differ by specific opioid [23]. In a review of nine systematic reviews of cancer pain patients (n=13,524), it was reported that 95% of cancer patients with moderate to severe pain who tolerate their prescribed opioids (including but not limited to tramadol) will have mild to no pain after about 14 days of treatment [24].

Tapentadol

For conventional analgesics, cLBP with a neuropathic component is a prevalent condition that remains challenging to treat. In a study comparing prolonged-release (PR) tapentadol monotherapy 500 mg to a combination treatment of PR tapentadol 300 mg plus pregabalin 300 mg, 288 patients with pain ≥6 at baseline demonstrated significant improvements in neuropathic pain relief and health-related quality of life at eight weeks, but the tapentadol monotherapy group had greater pain relief [25]. The rate of the composite adverse event of dizziness and/or somnolence was significantly lower in the PR tapentadol-only group than in the group taking PR tapentadol combined with pregabalin (16.9% vs. 27.0%, p=0.0302) [25]. In a sub-study of those patients with severe pain levels who responded well to PR tapentadol 300 mg, defined as having a pain score <4 during the titration phase, these responders were then administered PR tapentadol 300 mg/day over eight weeks in an open-label continuation study and the responders continued to experience significant improvements in neuropathic pain control, health-related quality of life, and overall pain control. Treatment-emergent adverse events occurred at a rate ≤5.1% in this study [26].

Long-term pain control can be challenging to offer as pain medications can become less effective over time or tolerability issues may arise. ER tapentadol was evaluated for use of three months or more in patients with moderate to severe musculoskeletal pain in a systematic review of four studies (n=4,094) [27]. ER tapentadol was found to reduce pain intensity when compared to placebo or the active comparator oxycodone and ER tapentadol had a more favorable tolerability profile than oxycodone [27]. Long-term efficacy of ER tapentadol was evaluated in a one-year, open-label extension study that followed several controlled trials (n=1,154) comparing ER tapentadol to oxycodone for knee pain associated with osteoarthritis or LBP [28]. Following dose titration, patients received ER tapentadol (100 to 200 mg twice daily) for a year. Mean pain at baseline was mild (3.9 on a 0-10 scale) and was maintained throughout the study (mean pain 3.7 at endpoint) [28].

These studies for cLBP, osteoarthritis, and musculoskeletal pain found that there was intersubject variability with respect to tapentadol response. Response stratification for any analgesic therapy is challenging but offers great potential to optimize pain control. In an open-label retrospective study of cLBP patients taking tapentadol, demographic characteristics failed to show any predictive value, but various metrics about pain intensity, pain characteristics, and the presence of neuropathic pain could be used to predict response, suggesting that response stratification algorithms might be possible [29].

Using the pain*DETECT* instrument to identify a neuropathic component in 196 severe cLBP patients, a 12-week open-label study was conducted using PR tapentadol, titrated to individual patients, but with a maximum daily dose of ≤500 mg with IR tapentadol 60 mg available for rescue analgesia during titration no more than twice daily [30]. In the maintenance phase, PR and IR tapentadol daily doses combined were ≤500 mg. In fact, at Week 6 of the study, the mean total daily dose (TDD) of tapentadol was 311.2 mg. Pain decreased 2.8 points on a 0-10 scale from baseline at 12 weeks overall. In patients with a neuropathic pain component, pain intensity decreased 3.0 compared to a decrease in 2.4 points in those without [30]. The pain*DETECT* instrument allows for a possible 38 point score if pain has a neuropathic component, so reduction of three points is not considered a substantial improvement.

Chronic neck pain is a prevalent condition, but there are a paucity of studies addressing appropriate treatment. In a study of 94 adults with severe neck pain refractory to step III opioid treatment, patients were administered 100 mg/day of PR tapentadol and then doses were titrated in 50 mg increments to a maximum dose of 500 mg/day. At the end of four weeks, 70% of patients had ≥30% improvement in pain and there were significant functional improvements as well based on the Neck Disability Index [31]. In a study of 54 patients with moderate to severe neck pain, ER tapentadol at doses starting at 100 mg/day and titrated (mean dose 204.5 mg/day) found that neuropathic pain decreased from being present in 70% of patients at baseline to 23% at 12 weeks. Neck Disability Index scores showed significant improvement and patients had significantly greater range of motion at 12 weeks [32].

Painful diabetic polyneuropathy is challenging to treat. A study of adults with moderate to severe pain associated with diabetic peripheral neuropathy allowed 318 patients to enter a three-week titration period when they received ER tapentadol 100 mg to 250 mg twice a day [33]. Those who responded, defined as achieving at least a one-point reduction in the 0-10 pain scale, then entered a 12-week study period where they were randomized to receive ER tapentadol at their optimal titrated dose or placebo. The average pain intensity at baseline was 7.33, decreasing to a mean of 4.16 at the end of the titration period (Week 3). At the end of 12 weeks, placebo patients had a mean pain score of 2.43 and ER tapentadol patients 2.04 (p<0.001) [33]. In a study of 24 patients with painful diabetic polyneuropathy, patients were randomized to sustained-release (SR) tapentadol (average dose 433 mg/day) or placebo for four weeks [34]. Prior to starting the study, patients were tested using two pain tests based on experimental pain: the conditioned pain modulation (CPM) and offset analgesia (OA). The CPM test measures endogenous pain modulation [35,36], while the OA test records the endogenous analgesic response to a noxious heat stimulus [37,38]. OA corresponds with activation of specific regions of the brain involved in the central modulation of pain [39]. No patient had significant CPM or OA results at baseline. After four weeks, CPM was activated significantly in the tapentadol group as these tapentadol patients experienced significant analgesic benefits. CPM changed from 9.1 at baseline to 4.3 (placebo patients) and to 24.2 (SR tapentadol patients), p<0.001. Likewise, pain relief was significantly superior in the SR tapentadol group than placebo (p=0.028). Neither placebo nor tapentadol had any effect on the OA scores [34]. Since descending pain pathways can modulate pain signals by inhibiting or amplifying them, this CMP-related effect may be of particular importance [40]. An imbalance between normal inhibition and amplification of pain signals may be at the root of many chronic painful conditions [41-44]. Following this line of reasoning, tapentadol would act first at the MORs and then inhibit neuronal norepinephrine reuptake [45] and, in that way, activate descending inhibitory pain pathways. However, CPM testing has known limitations: it is less effective in women and older adults, both of whom compose a large proportion of the chronic pain population [46,47]. On the other hand, OA occurs when pain processing is temporarily altered, resulting in a short-lived endogenous analgesic response and diabetic polyneuropathy patients may have dampened OA responses to begin with [37,38]. 

Tapentadol has been shown in some studies to be an effective analgesic for cancer pain, but prospective studies to date have been small [48]. In a systematic review and meta-analysis, cancer pain patients (n=1,029) taking tapentadol to manage cancer pain were evaluated and, although evidence was generally low quality, it was reported that tapentadol was similarly effective compared to oxycodone or morphine and with a similar rate of adverse events [49].

Cebranopadol

Cebranopadol is the newest of these agents and has not yet been cleared for market release. In a placebo-controlled study of patients with moderate to severe cLBP, cebranopadol was administered at doses of 200, 400, and 600 mcg with PR tapentadol 200 mg twice daily as comparator [50]. At 12 weeks, patient response, defined as ≥50% pain reduction, was achieved by 36.5%, 40.6%, and 38.9% of cebranopadol 200, 400, and 600 mcg patients, respectively, compared to 34.8% of PR tapentadol 200 mg twice daily patients and 27.5% of placebo patients. The total number of patients who reported a treatment-emergent adverse event were 68.2%, 60.2%, and 71.4% of cebranopadol 200, 400, and 600 mcg patients compared to 68.8% of PR tapentadol and 54.2% of placebo patients. The most frequently reported adverse events with cebranopadol were dizziness and nausea [50].

In a non-randomized open-label single-arm study, cancer patients with moderate to severe pain who had previously been treated in a double-blind clinical trial comparing PR morphine to cebranopadol entered a 26-week phase and were treated with cebranopadol at doses ranging from 200 to 1000 mcg per day [51]. Cebranopadol provided effective pain control [51].

Safety

Tramadol

Tolerance may develop with tramadol and its analgesic effect is limited as it is a weak opioid [2]. A retrospective study in Europe examined records of people ≥50 years with no history of hip fracture, cancer, or OUD, and created five sequential propensity-score-matched cohorts of patients who took tramadol, codeine, naproxen, ibuprofen, celecoxib, and etoricoxib. The primary endpoint was incident hip fracture at one year. In all cohorts, tramadol was associated with a higher risk of hip fracture than codeine or the non-steroidal anti-inflammatory drugs (NSAIDs) [52]. In a database study of 88,902 osteoarthritis patients ≥50 years, mortality rates were higher for tramadol patients than for patients taking diclofenac, naproxen, celecoxib, etoricoxib, or codeine. Tramadol was associated with higher all-cause mortality incidences than the NSAIDs but it was similar to that of codeine [53].

In an overview of Cochrane reviews about safety and tolerability of opioids (including but not limited to tramadol) for treating chronic non-cancer pain in adults, the absolute rate of any adverse event in placebo-controlled studies was 78% with an absolute event rate of 7.5% for a serious adverse event [54]. Tramadol has been associated with the classic opioid-related adverse events such as somnolence, nausea, vomiting, dizziness, constipation, pruritus, and others.

In a study of 21 healthy adult subjects taking both pregabalin and tramadol concurrently, no clinically relevant drug-drug interactions were reported and both drugs were well tolerated [55]. Patients received four-day courses of treatment: pregabalin 150 mg twice daily; ER tramadol 200 mg mornings and 100 mg evenings; concurrent use of 150 mg and 200 mg ER tramadol; and 150 mg pregabalin mornings and 100 mg ER tramadol evenings [55].

Tramadol has been associated with seizures but usually at supratherapeutic dose ranges [2]. Since cytochrome P-450 (CYP450) 2D6 metabolizes tramadol, there are phenotypic groups who appear to metabolize the drug extensively, ultra-rapidly, poorly, or somewhere in the middle [56]. The serotonin reuptake inhibition properties of tramadol are associated with seizures and tramadol should not be taken concurrently with selective serotonin reuptake inhibitors or tricyclic antidepressants, which may produce an overabundance of circulating serotonin resulting in serotonin toxicity or so-called “serotonin syndrome” [57]. These metabolizer categories may help explain why certain individuals and certain genetic groups are more prone to seizures. In a study of 1,480 men, tramadol-induced seizures occurred in 7% of patients (n=103) [58]. In preclinical studies, the dose of tramadol does not appear related to seizures, although in humans, only supratherapeutic doses of tramadol are associated with seizures [59]. In a study of 28 adults who had a history of tramadol use and seizures, tonic-clonic seizures occurred most often in the first 24 hours after drug intake and were more frequent when individuals were concurrently consuming alcohol, illicit drugs, antipsychotic drugs, or antidepressants [56].

Serotonin syndrome occurs as a result of build-up of serotonin (5-hydroxytryptamine or 5-HT) at the 5-HT receptors, capable of producing changes in mental status, autonomic hyperactivity, and neuromuscular symptoms. Other serotonin syndrome symptoms may include restlessness, anxiety, disorientation, clonus, muscle rigidity, tremor, hypertension, tachycardia, hyperthermia, and shivers [3]. Ranging in severity from mild to potentially fatal, serotonin syndrome has been observed in patients of all ages. The decarboxylation and hydroxylation of tryptophan produces 5-HT, which has binding affinity to at least seven different receptors, of which 5-HT1-A and 5-HT2-A are the most closely associated with serotonin syndrome [60]. Drugs such as tramadol that affect serotonin uptake, metabolism, synthesis, release, and other activities as well as drugs that may interfere with CYP450-2D6 and 3A4 metabolism can result in serotonin syndrome [3].

In the United States, the misuse and abuse of tramadol is less than that for hydrocodone and oxycodone [61]. Tramadol misuse is prevalent in other parts of the world, such as Iran [62] and parts of Africa [63]. In fact, tramadol is an emerging drug of abuse in many developing nations [64].

Tapentadol

In several comparative studies, tapentadol was found to be associated with adverse events similar to classic opioid-associated adverse events, but with a somewhat lower incidence of gastrointestinal side effects (nausea, vomiting, opioid-induced constipation) [65,66].

Using controlled-release (CR) oxycodone as the comparator drug, PR tapentadol was evaluated in a placebo-controlled clinical trial with 990 knee osteoarthritis patients [67]. After a three-week titration period, patients entered a 12-week maintenance period and the primary endpoint was the change in pain intensity score from baseline to the end of the maintenance period. The two active agents failed to achieve this endpoint (no significant decrease in pain). However, more PR tapentadol patients (58.3%) completed the study than oxycodone patients (36.6%) and tapentadol had a lower rate of constipation than oxycodone (17.9% vs. 35%) and nausea and/or vomiting (23.8% vs. 46.8%), p<0.001 for both [67].

In a study of 63 knee osteoarthritis patients with severe chronic pain administered PR tapentadol 50 mg to 250 mg twice daily over a five-week titration period followed by a seven-week maintenance period and using IR tapentadol as rescue analgesia, the response rate was 94.3% and pain decreased significantly from baseline to 12 weeks [68]. Adverse events decreased over time and 9.5% of patients withdrew from the study because of side effects. This study had to be terminated early because of poor enrollment and shortages of the study drug [68].

In a post-hoc pooled analysis of three randomized controlled trials of ER tapentadol (100 mg/day to 250 mg/day) for control of chronic knee pain associated with osteoarthritis, those patients with diagnosed hypertension prior to treatment (n=1,464) did not have clinically important changes in heart rate or blood pressure while taking ER tapentadol [69].

Tapentadol is not associated with serotonin syndrome but serotonin syndrome may occur when it is taken with other drugs that may lead to serotonin toxicity [70]. In fact, the opioid properties of tapentadol were believed to have masked the signs and symptoms of serotonin syndrome in one case report [71]. Tapentadol toxicity has been reported in the literature with such serious adverse events as respiratory depression, confusion, coma, seizures, and death, but these cases were not serotonin syndrome and the toxic potential of tapentadol is considered less than that of oxycodone or other typical opioids [72].

Tapentadol has a lower affinity for the MOR than other opioid analgesics, which suggests it has less abuse potential than typical opioids, such as morphine, hydromorphone, oxycodone, and others. In a retrospective study, it was found that the odds of abuse with tapentadol were 65% lower than those of oxycodone [73].

Cebranopadol

In the first human study of analgesic efficacy of cebranopadol, 641 cLBP patients were treated for 14 weeks with 200, 400, or 600 mcg of cebranopadol daily; tapentadol 200 mg twice daily; or placebo [50]. Treatment-emergent adverse events occurred in 80.4% of patients (83.1%, 84.3%, and 89.8% in the cebranopadol 200, 400, and 600 mcg arms, respectively) compared to 79.4% in the tapentadol and 65.1% in placebo groups [50]. The most frequently reported adverse events among cebranopadol patients were dizziness, nausea, somnolence, vomiting, constipation, fatigue, headache, and hyperhidrosis [50].

In a preclinical murine study, it was reported that the so-called “therapeutic window” between pain relief and respiratory depression was longer for cebranopadol than fentanyl, and this has been attributed to the NOP/FQ receptor activity of cebranopadol [74]. It has been suggested that cebranopadol has less potential for tolerance and dependence than typical opioids [75]. The abuse potential of cebranopadol was evaluated among 42 non-dependent recreational opioid users who were administered seven treatments (200, 400, 800 mcg of cebranopadol; 8 mg and 16 mg hydromorphone; and placebo) and measured “drug liking” on a visual analog scale at the point of peak effect [76]. Cebranopadol 200 mcg and 400 mcg had similar liking scores as placebo. At 800 mg, cebranopadol was “liked” similarly to hydromorphone 8 mg but less than hydromorphone 16 mg. No serious adverse events occurred [76].

In a non-randomized, open-label, 26-week clinical trial using oral cebranopadol to treat cancer pain, 84% of patients had at least one treatment-emergent adverse event and the most frequently observed were asthenia, malignant neoplasm progression, and reduced appetite [51].

Head-to-head clinical trials

Tramadol

An eight-week, randomized, double-blind, double-dummy, active-controlled, non-inferiority study (n=280) compared the safety and effectiveness of transdermal buprenorphine to SR tramadol in patients with moderate to severe musculoskeletal pain [77]. Patients were randomized to the buprenorphine group (5, 10, and 20 mcg/h patches, maximum dose 20 mcg/h) or SR tramadol 100 mg tablets, maximum 400 mg/day. Using the least squares mean difference in the change from baseline pain scores, the difference between the buprenorphine patch and tramadol arms were 0.45, which indicated that the patch was not inferior to SR tramadol. The rate of adverse events was similar between groups [77].

ER tramadol 200 mg was compared to modified-release (MR) flupirtine 400 mg and placebo in a four-week study of moderate to severe cLBP [78]. Flupirtine is a centrally acting analgesic agent that blocks glutamate N-methyl-D-aspartate (NMDA) receptors [79]. As such, flupirtine is neither an opioid nor a nonsteroidal anti-inflammatory drug. Flupirtine is approved in Europe, but not in the United States or Canada. This study found both treatments provided comparable pain control that was significantly superior to that of placebo. Significantly fewer flupirtine patients suffered from a treatment-emergent adverse event than the tramadol group (p=0.039) [78].

Tapentadol

Companion placebo-controlled studies compared IR tapentadol (50 or 75 mg) to IR oxycodone hydrochloride (10 mg) for efficacy in treating acute and chronic pain as well as tolerability [80]. Patients (n=589) had end-stage joint disease and were treated with the IR formulation for the first two weeks, then the ER formulation of the same drug or placebo for the next 28 days. Tapentadol had greater tolerability with respect to gastrointestinal symptoms and was similarly efficacious at relieving pain compared to oxycodone [80].

When compared to CR oxycodone, PR tapentadol over one year showed both agents provided adequate analgesia for moderate to severe knee or hip pain associated with osteoarthritis with similar rates of treatment-emergent adverse events (85.7% for tapentadol and 90.6% for oxycodone patients) [81]. More CR oxycodone patients discontinued the study treatment than PR tapentadol patients (36.8% vs. 22.1%, respectively) [81].

In a comparative trial of ER tapentadol (100 to 500 mg/day) or SR morphine (20 to 140 mg/day), cancer pain patients were all converted to ER tapentadol and 84.0% of patients maintained or had improved pain control in the first week of conversion [65]. Furthermore, gastrointestinal side effects were less with ER tapentadol than SR morphine [65].

In a study of cancer pain, 496 patients were randomized and then titrated to their optimal dose of PR tapentadol (100 to 250 mg twice daily) or CR morphine sulfate (40 to 100 mg twice daily) with rescue medication available in the form of IR morphine 10 mg (no maximum dose) [82]. Of the cohort, those who completed the four-week titration with a mean pain level of <5 in the final three days of titration and the mean use of rescue analgesics ≤20 mg/day then entered a four-week maintenance phase. Morphine patients continued on morphine, but PR tapentadol patients were randomized again with some remaining on PR tapentadol and others receiving placebo. Responders were defined as those who took 20 mg or less rescue medication a day, had a mean pain intensity of <5, and completed the maintenance portion of the trial. PR tapentadol patients had a response rate of 76.0% compared to 83.0% for morphine, which indicated that PR tapentadol was non-inferior to morphine. Treatment-emergent adverse events were higher with morphine (63.9%) than with PR tapentadol (50.0%) and placebo (56.3%) [82].

Cancer patients treated for pain (n=343) compared ER tapentadol to CR oxycodone and reported that tapentadol analgesia was non-inferior to that of oxycodone and the rate of adverse events (87.5% vs. 90.1% for tapentadol and oxycodone, respectively) were statistically similar [66]. However, tapentadol was associated with a lower rate of gastrointestinal side effects than oxycodone [66].

Cebranopadol

In a study of multiple doses of cebranopadol and PR morphine in 126 cancer patients, cebranopadol was noninferior to PR morphine for pain control and treatment-emergent adverse events were similar (83.1% for cebranopadol versus 82.0% for PR morphine) [13]. The dose ranges of cebranopadol in this study were 200 to 1,000 mcg per day [13].

Conversion of these three agents

In some situations, clinicians may wish to rotate patients from one of these single-entity molecules to another or from one formulation to another. The potential of converting IR tapentadol to ER tapentadol and vice versa was evaluated in a multicenter study of 88 patients with moderate to severe cLBP [83]. Patients were administered IR tapentadol 50, 75, or 100 mg every four to six hours up to a maximum TDD of 500 mg for three weeks to help identify the optimal daily dose for each individual. Patients were then randomized and entered a two-week double-blind phase in which they received their optimal IR tapentadol dose or ER tapentadol 100, 150, 200, or 250 mg that was deemed as close as possible to their optimal IR tapentadol dose. At two weeks, patients crossed over: IR tapentadol patients received ER tapentadol and vice versa. The mean pain intensity on a 0-10 scale during the final three days of each test phase served as the study endpoint. Pain intensity overall decreased from 7.3 at baseline to 4.2 after three weeks and remained relatively constant throughout the study. At the end of the study, IR tapentadol patients had a mean pain score of 3.9 while ER tapentadol patients had 4.0 (95% CI, -0.09 to 0.28) The median TDD of both formulations was 300 mg; rescue acetaminophen was used by 39.5% of IR and 45.2% of ER tapentadol groups. Treatment-emergent adverse events occurred at similar rates between groups. Thus, conversion of IR to ER and ER to IR tapentadol was safe, effective, and well tolerated [83].

Conversion from tramadol to tapentadol was reported in cancer patients who were transitioned directly to PR tapentadol from tramadol with ~70% achieving improved analgesic benefit with similar tolerability [84]. In a multicenter, controlled, double-blind study of 338 cancer patients, all patients were treated with tapentadol for pain control [84]. Of that cohort, 129/338 had been previously treated with tramadol within three days of randomization. Among responders, defined as those who achieved a pain rating of <5 on a 0 to 10 scale, the tramadol>tapentadol group had 69.8% versus the tapentadol group only, which had 63.9% responders. This study reported that cancer patients taking tramadol for pain could be effectively converted to tapentadol for pain control [84]. Non-cancer pain patients treated with tramadol but finding pain control inadequate can also be successfully transitioned to tapentadol [85].

In an open-label study of PR tapentadol (50 to 250 mg twice daily) for treatment of severe cLBP, patients were switched from previous typical opioid analgesic regimens to tapentadol, titrated over a period of five weeks, and then maintained for seven weeks [86]. Rescue analgesia was available in form of IR tapentadol 50 mg (up twice daily, four or more hours apart and not to exceed a TDD of PR and IR tapentadol of 500 mg/day). The endpoint was the percentage of patients who had a pain intensity score at Week 6 that was equal to or lower than their pain score at Week 1. The endpoint was met by 80.9% of subjects, significantly more than the null hypothesis (p<0.0001). This demonstrated PR tapentadol offered comparable if not better pain control than other opioid treatments [86].

## Conclusions

Tramadol, tapentadol, and cebranopadol may be considered atypical opioids in which dual mechanisms of action are present in a single molecule. These agents combine the analgesic efficacy of typical opioids with the promise of lesser abuse potential and greater tolerability. Their role in chronic pain control appears to be promising as they are effective pain relievers and appear to be at least as safe and tolerable as typical opioids. Tramadol is often used as first-line treatment of musculoskeletal pain while tapentadol is used for cancer pain, osteoarthritis, and other chronic pain conditions. Cebranopadol, which is not yet market released, is a first-in-class atypical opioid that has been effective in treating moderate to severe cLBP as well as cancer pain. These are important new agents in the analgesic armamentarium.

## References

[1] Coluzzi F, Mercadante S (2020). The potential role of dual mechanistic opioids in combating opioid misuse. Current Anesthesiology Reports.

[2] (2021). Tramadol. https://www.deadiversion.usdoj.gov/drug_chem_info/tramadol.pdf.

[3] Beakley BD, Kaye AM, Kaye AD (2015). Tramadol, pharmacology, side effects, and serotonin syndrome: a review. Pain Physician.

[4] Grond S, Sablotzki A (2004). Clinical pharmacology of tramadol. Clin Pharmacokinet.

[5] Fudin J, Boglish P (2021). Is tapentadol a glorified tramadol?. Pract Pain Manag.

[6] Chang EJ, Choi EJ, Kim KH (2016). Tapentadol: can it kill two birds with one stone without breaking windows?. Korean J Pain.

[7] Singh DR, Nag K, Shetti AN, Krishnaveni N (2013). Tapentadol hydrochloride: a novel analgesic. Saudi J Anaesth.

[8] Tzschentke TM, Jahnel U, Kogel B (2009). Tapentadol hydrochloride: a next-generation, centrally acting analgesic with two mechanisms of action in a single molecule. Drugs Today (Barc).

[9] Linz K, Christoph T, Tzschentke TM (2014). Cebranopadol: a novel potent analgesic nociceptin/orphanin FQ peptide and opioid receptor agonist. J Pharmacol Exp Ther.

[10] Raffa RB, Burdge G, Gambrah J (2017). Cebranopadol: novel dual opioid/NOP receptor agonist analgesic. J Clin Pharm Ther.

[11] Donica CL, Awwad HO, Thakker DR, Standifer KM (2013). Cellular mechanisms of nociceptin/orphanin FQ (N/OFQ) peptide (NOP) receptor regulation and heterologous regulation by N/OFQ. Mol Pharmacol.

[12] Nair AS, Mantha SP, Pulipaka SK, Rayani BK (2020). Cebranopadol: a first-in-class nociceptin receptor agonist for managing chronic pain. Indian J Palliat Care.

[13] Eerdekens MH, Kapanadze S, Koch ED, Kralidis G, Volkers G, Ahmedzai SH, Meissner W (2019). Cancer-related chronic pain: investigation of the novel analgesic drug candidate cebranopadol in a randomized, double-blind, noninferiority trial. Eur J Pain.

[14] Chidambaran V, Sadhasivam S (2019). Pharmacogenomics. A Practice of Anesthesia for Infants and Children (Sixth Edition).

[15] Schug SA (2007). The role of tramadol in current treatment strategies for musculoskeletal pain. Ther Clin Risk Manag.

[16] Babul N, Noveck R, Chipman H, Roth SH, Gana T, Albert K (2004). Efficacy and safety of extended-release, once-daily tramadol in chronic pain: a randomized 12-week clinical trial in osteoarthritis of the knee. J Pain Symptom Manage.

[17] Duehmke RM, Derry S, Wiffen PJ, Bell RF, Aldington D, Moore RA (2017). Tramadol for neuropathic pain in adults. Cochrane Database Syst Rev.

[18] Toupin April K, Bisaillon J, Welch V (2019). Tramadol for osteoarthritis. Cochrane Database Syst Rev.

[19] Chaparro LE, Furlan AD, Deshpande A, Mailis-Gagnon A, Atlas S, Turk DC (2013). Opioids compared to placebo or other treatments for chronic low-back pain. Cochrane Database Syst Rev.

[20] Whittle SL, Richards BL, Husni E, Buchbinder R (2011). Opioid therapy for treating rheumatoid arthritis pain. Cochrane Database Syst Rev.

[21] Wiffen PJ, Derry S, Moore RA (2017). Tramadol with or without paracetamol (acetaminophen) for cancer pain. Cochrane Database Syst Rev.

[22] Prommer EE (2005). Tramadol: does it have a role in cancer pain management?. J Opioid Manag.

[23] Boland JW, Pockley AG (2018). Influence of opioids on immune function in patients with cancer pain: from bench to bedside. Br J Pharmacol.

[24] Wiffen PJ, Wee B, Derry S, Bell RF, Moore RA (2017). Opioids for cancer pain - an overview of Cochrane reviews. Cochrane Database Syst Rev.

[25] Baron R, Martin-Mola E, Müller M, Dubois C, Falke D, Steigerwald I (2015). Effectiveness and safety of tapentadol prolonged release (PR) versus a combination of tapentadol PR and pregabalin for the management of severe, chronic low back pain with a neuropathic component: a randomized, double-blind, phase 3b study. Pain Pract.

[26] Baron R, Kern U, Müller M, Dubois C, Falke D, Steigerwald I (2015). Effectiveness and tolerability of a moderate dose of tapentadol prolonged release for managing severe, chronic low back pain with a neuropathic component: an open-label continuation arm of a randomized phase 3b study. Pain Pract.

[27] Santos J, Alarcão J, Fareleira F, Vaz-Carneiro A, Costa J (2015). Tapentadol for chronic musculoskeletal pain in adults. Cochrane Database Syst Rev.

[28] Buynak R, Rappaport SA, Rod K, Arsenault P, Heisig F, Rauschkolb C, Etropolski M (2015). Long-term safety and efficacy of tapentadol extended release following up to 2 years of treatment in patients with moderate to severe, chronic pain: results of an open-label extension trial. Clin Ther.

[29] Reimer M, Hüllemann P, Hukauf M, Keller T, Binder A, Gierthmühlen J, Baron R (2017). Prediction of response to tapentadol in chronic low back pain. Eur J Pain.

[30] Steigerwald I, Müller M, Davies A (2012). Effectiveness and safety of tapentadol prolonged release for severe, chronic low back pain with or without a neuropathic pain component: results of an open-label, phase 3b study. Curr Med Res Opin.

[31] Coluzzi F, Pergolizzi JV Jr, Giordan E, Locarini P, Boaro A, Billeci D (2020). Tapentadol prolonged release for managing moderate to severe chronic neck pain with or without a neuropathic component. Curr Med Res Opin.

[32] Billeci D, Coluzzi F (2017). Tapentadol extended release for the management of chronic neck pain. J Pain Res.

[33] Vinik AI, Shapiro DY, Rauschkolb C, Lange B, Karcher K, Pennett D, Etropolski MS (2014). A randomized withdrawal, placebo-controlled study evaluating the efficacy and tolerability of tapentadol extended release in patients with chronic painful diabetic peripheral neuropathy. Diabetes Care.

[34] Niesters M, Proto PL, Aarts L, Sarton EY, Drewes AM, Dahan A (2014). Tapentadol potentiates descending pain inhibition in chronic pain patients with diabetic polyneuropathy. Br J Anaesth.

[35] Arendt-Nielsen L, Andresen T, Malver LP, Oksche A, Mansikka H, Drewes AM (2012). A double-blind, placebo-controlled study on the effect of buprenorphine and fentanyl on descending pain modulation: a human experimental study. Clin J Pain.

[36] Yarnitsky D, Arendt-Nielsen L, Bouhassira D (2010). Recommendations on terminology and practice of psychophysical DNIC testing. Eur J Pain.

[37] Grill JD, Coghill RC (2002). Transient analgesia evoked by noxious stimulus offset. J Neurophysiol.

[38] Niesters M, Hoitsma E, Sarton E, Aarts L, Dahan A (2011). Offset analgesia in neuropathic pain patients and effect of treatment with morphine and ketamine. Anesthesiology.

[39] Derbyshire SW, Osborn J (2009). Offset analgesia is mediated by activation in the region of the periaqueductal grey and rostral ventromedial medulla. Neuroimage.

[40] Ossipov MH, Dussor GO, Porreca F (2010). Central modulation of pain. J Clin Invest.

[41] King CD, Wong F, Currie T, Mauderli AP, Fillingim RB, Riley JL 3rd (2009). Deficiency in endogenous modulation of prolonged heat pain in patients with irritable bowel syndrome and temporomandibular disorder. Pain.

[42] Lautenbacher S, Rollman GB (1997). Possible deficiencies of pain modulation in fibromyalgia. Clin J Pain.

[43] Olesen SS, Brock C, Krarup AL, Funch-Jensen P, Arendt-Nielsen L, Wilder-Smith OH, Drewes AM (2010). Descending inhibitory pain modulation is impaired in patients with chronic pancreatitis. Clin Gastroenterol Hepatol.

[44] Seifert F, Kiefer G, DeCol R, Schmelz M, Maihöfner C (2009). Differential endogenous pain modulation in complex-regional pain syndrome. Brain.

[45] Kress HG (2010). Tapentadol and its two mechanisms of action: is there a new pharmacological class of centrally-acting analgesics on the horizon?. Eur J Pain.

[46] Edwards RR, Fillingim RB, Ness TJ (2003). Age-related differences in endogenous pain modulation: a comparison of diffuse noxious inhibitory controls in healthy older and younger adults. Pain.

[47] Lautenbacher S, Kunz M, Burkhardt S (2008). The effects of DNIC-type inhibition on temporal summation compared to single pulse processing: does sex matter?. Pain.

[48] Carmona-Bayonas A, Jiménez Fonseca P, Virizuela Echaburu J (2017). Tapentadol for cancer pain management: a narrative review. Pain Pract.

[49] Wiffen PJ, Derry S, Naessens K, Bell RF (2015). Oral tapentadol for cancer pain. Cochrane Database Syst Rev.

[50] Christoph A, Eerdekens MH, Kok M, Volkers G, Freynhagen R (2017). Cebranopadol, a novel first-in-class analgesic drug candidate: first experience in patients with chronic low back pain in a randomized clinical trial. Pain.

[51] Koch ED, Kapanadze S, Eerdekens MH, Kralidis G, Létal J, Sabatschus I, Ahmedzai SH (2019). Cebranopadol, a novel first-in-class analgesic drug candidate: first experience with cancer-related pain for up to 26 weeks. J Pain Symptom Manage.

[52] Wei J, Lane NE, Bolster MB (2020). Association of tramadol use with risk of hip fracture. J Bone Miner Res.

[53] Zeng C, Dubreuil M, LaRochelle MR (2019). Association of tramadol with all-cause mortality among patients with osteoarthritis. JAMA.

[54] Els C, Jackson TD, Kunyk D (2017). Adverse events associated with medium- and long-term use of opioids for chronic non-cancer pain: an overview of Cochrane Reviews. Cochrane Database Syst Rev.

[55] Lee S, Kim Y, Lee JJ, Im G, Cho JY, Chung JY, Yoon S (2018). A pharmacokinetic drug-drug interaction study between pregabalin and tramadol in healthy volunteers. Eur J Clin Pharmacol.

[56] Boostani R, Derakhshan S (2012). Tramadol induced seizure: a 3-year study. Caspian J Intern Med.

[57] Mason BJ, Blackburn KH (1997). Possible serotonin syndrome associated with tramadol and sertraline coadministration. Ann Pharmacother.

[58] Shamloul RM, Elfayomy NM, Ali EI, Elmansy AM, Farrag MA (2020). Tramadol-associated seizures in Egypt: Epidemiological, clinical, and radiological study. Neurotoxicology.

[59] Beyaz SG, Sonbahar T, Bayar F, Erdem AF (2016). Seizures associated with low-dose tramadol for chronic pain treatment. Anesth Essays Res.

[60] Mills KC (1995). Serotonin syndrome. Am Fam Physician.

[61] Reines SA, Goldmann B, Harnett M, Lu L (2020). Misuse of tramadol in the United States: an analysis of the national survey of drug use and health 2002-2017. Subst Abuse.

[62] Rostam-Abadi Y, Gholami J, Amin-Esmaeili M (2020). Tramadol use and public health consequences in Iran: a systematic review and meta-analysis. Addiction.

[63] Saapiire F, Namillah G, Tanye V, Abubakari A (2021). The insurgence of tramadol abuse among the most active population in Jirapa municipality: a study to assess the magnitude of the abuse and its contributory factors. Psychiatry J.

[64] Roa JA, Guevara A, Guevara C, Guevara-Aguirre J (2019). Physician's role in prescribing opioids in developing countries. BMJ Case Rep.

[65] Imanaka K, Tominaga Y, Etropolski M, Ohashi H, Hirose K, Matsumura T (2014). Ready conversion of patients with well-controlled, moderate to severe, chronic malignant tumor-related pain on other opioids to tapentadol extended release. Clin Drug Investig.

[66] Imanaka K, Tominaga Y, Etropolski M (2013). Efficacy and safety of oral tapentadol extended release in Japanese and Korean patients with moderate to severe, chronic malignant tumor-related pain. Curr Med Res Opin.

[67] Serrie A, Lange B, Steup A (2017). Tapentadol prolonged-release for moderate-to-severe chronic osteoarthritis knee pain: a double-blind, randomized, placebo- and oxycodone controlled release-controlled study. Curr Med Res Opin.

[68] Steigerwald I, Schenk M, Lahne U, Gebuhr P, Falke D, Hoggart B (2013). Effectiveness and tolerability of tapentadol prolonged release compared with prior opioid therapy for the management of severe, chronic osteoarthritis pain. Clin Drug Investig.

[69] Biondi DM, Xiang J, Etropolski M, Moskovitz B (2014). Evaluation of blood pressure and heart rate in patients with hypertension who received tapentadol extended release for chronic pain: a post hoc, pooled data analysis. Clin Drug Investig.

[70] Gressler LE, Hammond DA, Painter JT (2017). Serotonin syndrome in tapentadol literature: systematic review of original research. J Pain Palliat Care Pharmacother.

[71] Walczyk H, Liu CH, Alafris A, Cohen H (2016). Probable tapentadol-associated serotonin syndrome after overdose. Hosp Pharm.

[72] Channell JS, Schug S (2018). Toxicity of tapentadol: a systematic review. Pain Manag.

[73] Cepeda MS, Fife D, Ma Q, Ryan PB (2013). Comparison of the risks of opioid abuse or dependence between tapentadol and oxycodone: results from a cohort study. J Pain.

[74] Linz K, Schröder W, Frosch S, Christoph T (2017). Opioid-type respiratory depressant side effects of cebranopadol in rats are limited by its nociceptin/orphanin FQ peptide receptor agonist activity. Anesthesiology.

[75] Atkinson T, Cowley H (2021). Analgesics of the future: cebranopadol as an opioid alternative. Practical Pain Management.

[76] Göhler K, Sokolowska M, Schoedel KA, Nemeth R, Kleideiter E, Szeto I, Eerdekens MH (2019). Assessment of the abuse potential of cebranopadol in nondependent recreational opioid users: a phase 1 randomized controlled study. J Clin Psychopharmacol.

[77] Leng X, Li Z, Lv H (2015). Effectiveness and safety of transdermal buprenorphine versus sustained-release tramadol in patients with moderate to severe musculoskeletal pain: an 8-week, randomized, double-blind, double-dummy, multicenter, active-controlled, noninferiority study. Clin J Pain.

[78] Uberall MA, Mueller-Schwefe GH, Terhaag B (2012). Efficacy and safety of flupirtine modified release for the management of moderate to severe chronic low back pain: results of SUPREME, a prospective randomized, double-blind, placebo- and active-controlled parallel-group phase IV study. Curr Med Res Opin.

[79] Harish S, Bhuvana K, Bengalorkar GM, Kumar T (2012). Flupirtine: clinical pharmacology. J Anaesthesiol Clin Pharmacol.

[80] Etropolski M, Kelly K, Okamoto A, Rauschkolb C (2011). Comparable efficacy and superior gastrointestinal tolerability (nausea, vomiting, constipation) of tapentadol compared with oxycodone hydrochloride. Adv Ther.

[81] Wild JE, Grond S, Kuperwasser B (2010). Long-term safety and tolerability of tapentadol extended release for the management of chronic low back pain or osteoarthritis pain. Pain Pract.

[82] Kress HG, Koch ED, Kosturski H (2014). Tapentadol prolonged release for managing moderate to severe, chronic malignant tumor-related pain. Pain Physician.

[83] Etropolski MS, Okamoto A, Shapiro DY, Rauschkolb C (2010). Dose conversion between tapentadol immediate and extended release for low back pain. Pain Physician.

[84] Kress HG, Koch ED, Kosturski H (2016). Direct conversion from tramadol to tapentadol prolonged release for moderate to severe, chronic malignant tumour-related pain. Eur J Pain.

[85] Richter U, Waldmann-Rex S, Lehmann U (2015). Conversion to tapentadol PR improves analgesia and quality of life in patients with severe and chronic pain despite using tramadol > 300 mg/d. (Article in German). Wien Klin Wochenschr.

[86] Gálvez R, Schäfer M, Hans G, Falke D, Steigerwald I (2013). Tapentadol prolonged release versus strong opioids for severe, chronic low back pain: results of an open-label, phase 3b study. Adv Ther.

